# Examination of polymorphic glutathione S-transferase (GST) genes, tobacco smoking and prostate cancer risk among Men of African Descent: A case-control study

**DOI:** 10.1186/1471-2407-9-397

**Published:** 2009-11-16

**Authors:** Nicole A Lavender, Marnita L Benford, Tiva T VanCleave, Guy N Brock, Rick A Kittles, Jason H Moore, David W Hein, La Creis R Kidd

**Affiliations:** 1 Department of Pharmacology & Toxicology, University of Louisville (UofL), School of Medicine, 500 South Preston Street, Room 1319 Research Tower, UofL Health Science Center, Louisville, KY 40202, USA; 2 James Graham Brown Cancer Center, Cancer Prevention & Control Program, UofL, 529 South Jackson Street, Louisville, KY 40202, USA; 3 Department of Bioinformatics & Biostatistics, UofL School of Public Health and Information Sciences, 485 East Gray Street, Louisville, KY 40202, USA; 4 Center for Genetics and Molecular Medicine, UofL School of Medicine, Delia B. Baxter Biomedical Research Building, Suite 221, Louisville, Kentucky 40202, USA; 5 Center for Environmental Genomics and Integrative Biology, UofL, Delia B. Baxter Biomedical Research Building, Suite 221, Louisville, Kentucky 40202, USA; 6 Department of Medicine, University of Chicago, 5841 S. Maryland Ave, MC Chicago, Illinois 606037, USA; 7 Dartmouth -Hitchcock Medical Center, Dartmouth Medical School, 706 Rubin Building, HB 7937 One, Medical Center Drive, Dartmouth-Hitchcock Medical Center Lebanon, NH 03756, USA; 8 Department of Epidemiology and Public Health, UofL School of Public Health and Information Sciences, 485 East Gray Street, Louisville, KY 40202, USA

## Abstract

**Background:**

Polymorphisms in *glutathione S-transferase *(GST) genes may influence response to oxidative stress and modify prostate cancer (PCA) susceptibility. These enzymes generally detoxify endogenous and exogenous agents, but also participate in the activation and inactivation of oxidative metabolites that may contribute to PCA development. Genetic variations within selected *GST *genes may influence PCA risk following exposure to carcinogen compounds found in cigarette smoke and decreased the ability to detoxify them. Thus, we evaluated the effects of polymorphic *GSTs *(*M1*, *T1*, and *P1*) alone and combined with cigarette smoking on PCA susceptibility.

**Methods:**

In order to evaluate the effects of *GST *polymorphisms in relation to PCA risk, we used TaqMan allelic discrimination assays along with a multi-faceted statistical strategy involving conventional and advanced statistical methodologies (e.g., Multifactor Dimensionality Reduction and Interaction Graphs). Genetic profiles collected from 873 men of African-descent (208 cases and 665 controls) were utilized to systematically evaluate the single and joint modifying effects of *GSTM1 *and *GSTT1 *gene deletions, *GSTP1 *105 Val and cigarette smoking on PCA risk.

**Results:**

We observed a moderately significant association between risk among men possessing at least one variant *GSTP1 *105 Val allele (OR = 1.56; 95%CI = 0.95-2.58; p = 0.049), which was confirmed by MDR permutation testing (p = 0.001). We did not observe any significant single gene effects among *GSTM1 *(OR = 1.08; 95%CI = 0.65-1.82; p = 0.718) and *GSTT1 *(OR = 1.15; 95%CI = 0.66-2.02; p = 0.622) on PCA risk among all subjects. Although the *GSTM1*-*GSTP1 *pairwise combination was selected as the best two factor LR and MDR models (p = 0.01), assessment of the hierarchical entropy graph suggested that the observed synergistic effect was primarily driven by the *GSTP1 *Val marker. Notably, the *GSTM1*-*GSTP1 *axis did not provide additional information gain when compared to either loci alone based on a hierarchical entropy algorithm and graph. Smoking status did not significantly modify the relationship between the *GST *SNPs and PCA.

**Conclusion:**

A moderately significant association was observed between PCA risk and men possessing at least one variant *GSTP1 *105 Val allele (p = 0.049) among men of African descent. We also observed a 2.1-fold increase in PCA risk associated with men possessing the *GSTP1 *(Val/Val) and *GSTM1 *(*1/*1 + *1/*0) alleles. MDR analysis validated these findings; detecting *GSTP1 *105 Val (p = 0.001) as the best single factor for predicting PCA risk. Our findings emphasize the importance of utilizing a combination of traditional and advanced statistical tools to identify and validate single gene and multi-locus interactions in relation to cancer susceptibility.

## Background

Even though prostate cancer (PCA) ranks the highest in incidence and second mortality among all cancers affecting American men, its etiology and ethnic disparities are largely unknown[1]. For instance, age and family history are the strongest risk factors for PCA, but African-American (AA) men are more than twice as likely to develop the disease compared to other racial or ethnic groups[1]. AA men are also more likely to be diagnosed with PCA at a younger age, with more aggressive disease and poorer prognosis [1-3]. Despite increases in five year survival rates for AA men over the last few decades; their rates still lag far behind other races[1]. While, the reasons for this disparity are largely speculative; risk, incidence, and mortality rates suggest that genetic factors play an important role in PCA initiation and progression[1]. However, lifestyle habits (e.g., cigarette smoking, diet) have also been implicated to increase risk; indicating that environmental factors may contribute to PCA[1,2,4]. Therefore, prostate carcinogenesis and its disparity most likely involve a complex interplay between genetic and environmental factors. More specifically, variations in carcinogen metabolism genes may play a critical role in PCA development due to their activation or detoxification functions.

The *glutathione S-transferase *(*GST*) gene superfamily encodes enzymes that catalyze the conjugation of glutathione to electrophilic compounds[5,6]. These enzymes generally detoxify endogenous and exogenous agents, but also participate in the activation and inactivation of oxidative metabolites of carcinogenic compounds associated with prostate cancer[7,8]. Variant *GST *alleles have been identified within the general population. The most extensively studied variant *GST*s include two *GST *deletion alleles (i.e., GSTM1*0/*0 [GenBank: BC024005.2] and GSTT1*0/*0 [GenBank: BC007065.1]) and the *GSTP1 *Val allele which is characterized by an adenine to guanine substitution at position -313 (A^-313^G) in exon 5 [GenBank: BC010915.1; dbSNP: rs1695][9]. The functional consequences of the *GSTM1 *and *GSTT1 *(*0/*0) genotypes are obvious in terms of enzyme activity; gene deletion results in loss of conjugation potential. The *GSTP1 *polymorphism, resulting from an isoleucine to valine substitution within the active site of the enzyme at codon 105 (I^105^V), is linked to altered substrate-specific thermostability and conjugation activity [10-12]. For instance, the *GSTP1 *105 Val variant has been associated with lower efficiency for diol epoxides of some polycyclic aromatic hydrocarbons, therefore resulting in decreased detoxification of these compounds compared to the Ile allele[10].

Genetic variations in polymorphic *GST *genes have been implicated in the etiology of numerous cancers [13-18]. Some studies indicate *GST *polymorphisms are associated with prostate cancer; however, this association is not accepted across all observational studies [18-25]. Discrepancies may be partially attributed to failure to consider gene combinations or interactions with tobacco smoking. Furthermore, few studies have involved a sufficient number of African-Americans in order to adequately investigate the role of *GST *polymorphisms and environmental factors in PCA development[12,26,27].

To clarify the role of *GST *genes in PCA risk within a high-risk sub-group, we evaluated the individual and joint modifying effects of three commonly studied sequence variants in a case-control study of 208 cases and 665 disease-free controls among a population of men of African descent. In an exploratory analysis, we evaluated gene-gene interactions using an available non-parametric statistical model, namely Multifactor Dimensionality Reduction (MDR). This advanced statistical tool readily overcomes sample size limitations often encountered by parametric statistical methods (e.g., logistic regression analysis). MDR has greater than 80% statistical power and rigor to evaluate gene-gene interactions even in the presence of small sample sizes (i.e., 200 cases and 200 controls). This computationally sound statistical tool enabled evaluations of single and multi-locus genetic and environmental markers as indicators of PCA susceptibility within our population sample.

## Methods

### Study population

Unrelated male residents (n = 1016) of Washington D.C. and Columbia SC, were considered for eligibility in the current PCA case control study. Study participants (n = 132) were not considered in the current study if they met one or more of the following exclusion criteria: (1) they were diagnosed with benign prostatic hyperplasia (n = 64); (2) had an abnormal prostate specific antigen (PSA) and digital rectal examination (DRE) (n = 11); and (3) had European ancestry based on a Global Ancestry score of < 25% (n = 70)[28]. Eligible men of African descent (i.e., self-identified African-Americans, East Africans, West Africans, and Afro-Caribbeans), including 208 patients (ages 41-91) and 665 healthy volunteers (ages 26-89), were recruited from the Howard University Hospital (HUH) Division of Urology PCA patient population, the HUH PCA screening program, and the South Carolina PCA screening program (Table 1). The PCA patients and screening participants, recruited between 2001 and 2005, had a PSA and DRE. Subjects who had an abnormal PSA (>4.0 ng/ml) and/or irregular DRE underwent multiple core needle biopsies. Incident PCA cases in the current study were identified by an HUH urologist based on abnormal PSA (>2.5 ng/ml) and/or DRE as well as histological findings following a radical prostatectomy. Inclusion criteria of controls were men with PSA levels ≤ 2.5 ng/ml and/or normal DREs/biopsies. Tumor grade, ranging from 4-10, was collected for 62.0% of the cases (n = 129). Subjects (n = 216) were classified as current (n = 38), former (n = 71) and never (n = 107) smokers. All study participants had available DNA extracted from whole blood and provided written informed consent for participation in genetic analysis studies under a protocol approved by the Howard University Institutional Review Board as well as from the HUH Division of Urology.

**Table 1:** Patient and Tumor Characteristics

Characteristics	Cases	Controls	p-value^a^
**Number of Participants, n**	208	665	---

**Age (yrs)**			
Median (range)	65 (41-91)	52 (26-89)	0.0001

**PSA in ng/ml **n (%)			
< 4	43 (22.0)	609 (94.4)	< 0.0001
≥ 4	152 (78.0)	36 (5.6)	

**Gleason Score **n (%)			
4	17 (13.2)		
5	15 (11.6)		
6	33 (25.6)		
7	40 (31.0)		
8	6 (4.6)		
9	14 (10.9)		
10	4 (3.10)		

**Smokers **n (%)			
Never	62 (45.6)	45 (20.8)	0.162
Former	51 (37.5)	20 (25.0)	
Current	23 (16.9)	15 (18.8)	

**Eversmokers **n (%)			
No	62 (45.6)	45 (56.3)	0.130
Yes	74 (54.4)	35 (43.8)	

**Global West African Ancestry** **Median (SD)**	0.791 (0.253-0.947)	0.718 (0.255-0.946)	0.020

Abbreviations: PSA, prostate specific antigen

^a^Differences in frequencies were tested by a Chi-square test of heterogeneity (i.e., PSA ng/ml); Differences in age and Global West African Ancestry between cases and controls were tested using the Wilcoxon sum Rank test.

### TaqMan allelic discrimination of GSTM1, GSTP1 and GSTT1 sequence variants

Polymorphisms in three *glutathione S-transferases *genes were ascertained using TaqMan Polymerase Chain Reaction (PCR) allelic discrimination assays. The following alleles were detected: (1) *GSTM1 *(*1/*1 + *1/*0); (2) *GSTT1 *(*1/*0 + *1/*0); and (3) *GSTP1 *(Ile105Val). The albumin reference gene served as a positive control for *GST *(*M1*, *T1*) deletions. The discrimination assay contained approximately 40 ng of germ-line DNA, 1× Universal Master Mix (Applied Biosystems), 900 nM of each primer (forward and reverse), and 400 nM of each probe (FAM and VIC) to comprise a 10 μl reaction. The primers and probes used to detect a deletion or single nucleotide polymorphisms (SNP) detected in *GSTM1*, *GSTP1 *and *GSTT1 *alleles were obtained using the NCI SNP500 database, published reports and Primer Express 3.0 software (Applied Biosystems, Foster City, CA) [29-31]. The PCR amplification conditions consisted of the following: an initial 2 step hold (50°C for 2 min, followed by 95°C for 10 min) and 40 cycles of a two-step PCR (95°C for 15 s, 60°C for 1 min)[30]. The fluorescent intensity emitted from the fluorogenic probes were measured using the ABI 7900HT sequence detection system and assigned genotypes using SDS 2.2.1 software (Applied Biosystems, Foster City, CA). To minimize misclassification bias, laboratory technicians were blinded to the case status of study participants. Based on 24 non-DNA template controls per batch analysis, percent cross-contamination during sample handling was negligent (<0%). Duplicate genotyping performed on 72 randomly selected samples for quality control purposes resulted in concordance rates of 96-97% for *GST *(*M1*, *T1*) and 100% for *GSTP1*. Call rates were >94% for the *GST *deletion (*M1*, *T1*) and *GSTP1 *allelic discrimination assays. In addition, we tested whether the aforementioned *GST *polymorphisms were in Hardy-Weinberg equilibrium among controls using a significance level of p < 0.05.

### Ancestry markers

One hundred previously validated autosomal ancestry markers were included to account for potential population stratification among our admixed population of self-reported African-Americans, West African, East African, Afro-Caribbean, as previously described[32]. Study participants were grouped from lowest to highest genetic West African Ancestry (WAA), with scores ranging from 0-100%. These 100 markers were assembled using DNA from self-identified African-Americans (Coriell Institute for Medical Research, n = 96), Yoruban West Africans (HapMap, n = 60), West Africans (Bantu and Nilo Saharan speakers, n = 72), Europeans (New York City, n = 24), and CEPH Europeans (HapMap Panel, n = 60), as previously reported[32]. Individuals (n = 873) with a high degree of WAA greater than or equal to 25% were considered in the final analysis.

### Evaluation of individual GST loci and PCA risk using LR analysis

To assess whether inheritance of at least one *GST *(*M1*, *T1*) deletion or *GSTP1 *Val allele was associated with elevated risk of developing PCA, we tested for significant differences in the distribution of variant genotypes between 208 cases and 665 controls using the chi-square test of homogeneity. Associations between PCA risk and candidate polymorphic genes, expressed as odds ratios (ORs) and corresponding 95% confidence intervals (CIs), were estimated using unconditional multivariate logistic regression (LR) models adjusted for potential confounders [age (yrs), PSA (ng/ml), and WAA (continuous)]. All reported risk estimates and 95% CIs for the selected polymorphic *GST *genes used the following as reference genotypes: *GSTM1 *(*1/*1 + *1/*0), *GSTT1 *(*1/*1 + *1/*0), *GSTP1 *(Ile/Ile). Test for trend included genotypes as ordinal variables. Statistical significance was assessed using a p-value < 0.05. All chi-square test and LR analyses were conducted using SAS 9.1.3 (SAS Institute, Cary, NC).

### Gene combination effects

MDR was used to evaluate gene-gene interactions associated with PCA risk. This tool aids in the identification of high-risk markers using a cross validation strategy to estimate the classification and prediction accuracy of individual and multifactor models [33-35]. MDR is a data mining platform that readily overcomes sample size limitations often encountered by parametric statistical methods (e.g., LR analysis) by collapsing high-dimensional genetic data into a single dimension [33-35]. Also, MDR is a "model-free" (it does not assume a specified genetic model) and "non-parametric" method (it does not estimate parameters) that is effective with relatively small sample sizes [33-35]. With MDR, we pooled multi-locus factors into high-risk and low-risk groups, thereby reducing the otherwise high-dimensional data to a single variable and permitting an investigation of individual *GST *genes, gene combination effects, and *GST*-tobacco smoking interactions. This one-dimensional multi-locus variable was evaluated for its ability to classify and predict PCA susceptibility through cross-validation and permutation testing. MDR was utilized to generate a single model that maximizes the number of individuals with the proper risk assignment. Single best factor models were selected among each of the one- or two- factor combinations based on those that minimize the prediction error as well as maximize cross validation consistency (CVC) and average testing accuracy (ATA). To evaluate the number of times the same individual loci or set of genes was identified in each possible 9/10^ths ^of the data, the average CVC (based on a scale from 0-100%) from the observed data was compared to the distribution of average consistencies under the null hypothesis of no association. Statistically significant single and multi-locus models may be adjusted for potential confounders by placing the MDR output variables into a LR model. Furthermore, validation of models as effective predictors of prostate cancer susceptibility was derived empirically from 1,000 permutations. This approach accounted for multiple testing issues as long as the entire model fitting procedure was repeated for each randomized dataset to provide an opportunity to identify false-positives. We considered MDR permutation results to be statistically significant at the 0.05 level. LR analysis was used to perform two-way interaction models for risk estimates terms identified by MDR.

### Interaction Entropy Graphs

Interaction entropy was used as a third strategy to verify, visualize, and interpret combination effects identified by LR and MDR [36,37]. Interaction entropy uses information gain (IG) to gauge whether interactions between two or more variables provide more information about a class variable relative to each variable considered independently [36] and has been applied to several recent epidemiological studies [34,36,38,39]. The colors range from red representing a high degree of synergy (positive information gain), orange a lesser degree, and gold representing independence and a midway point between synergy and redundancy. Blue represents the highest level of redundancy (negative information gain), followed by green.

## Results

The patient and tumor characteristics in the current study are summarized in Table 1. Cases were significantly older than controls and had higher PSA levels. Although there was a small portion of controls (5.6%) who had PSA levels that exceeded 4.0 ng/ml, these individuals did not have an abnormal DRE or irregular biopsy. There was a significant difference in median West African genetic ancestry estimates comparing cases and controls (p = 0.020). Cigarette smoking data was available for approximately 25% of our study subjects (n = 216, 136 cases and 80 controls). These subjects were classified by cigarette smoking: current (n = 38), former (n = 71) and never (n = 107). Cigarette smoking did not differ significantly (p > 0.05) between cases and controls.

Within our study set, inheritance of two *GST *(*M1*, *T1*) deletions or at least one *GSTP1 *105 Val allele was fairly common among controls with frequencies ranging from 17.5-47.9%, as detailed in Table 2. The genotype frequencies among controls were comparable to those observed in other African descendent sub-groups[26,40,41]. The genotype frequencies among controls did not deviate from expected distributions based on the Hardy-Weinberg equilibrium (p ≥ 0.138).

**Table 2:** Association between prostate cancer risk and selected *GST *gene variations

Gene	SNP	Case n (%)	Control n (%)	Risk Estimate^†^ OR (95% CI)	Risk Estimate^††^ Adj OR (95% CI)	p-value^†††^	p-value for Trend
*GSTP1*	AA (Ile/Ile)	55 (29.0)	186 (32.5)	1.00 (Reference)	1.00 (Reference)		
A^313^G	AG (Ile/Val)	85 (44.7)	274 (47.9)	1.12 (0.77-1.63)	1.09 (0.67-1.81)		
Ile^105^Val	GG(Val/Val)	50 (26.3)	112 (19.6)	1.62 (1.04-2.51)	1.66 (0.94-2.96)	0.141	0.080
	AG+GG	140 (73.7)	460 (80.4)	1.47 (1.00-2.15)	1.56 (0.95-2.58)	0.049	
	missing	6 (3.2)	35 (6.1)				

*GSTM1*	*1/*1 + *1/*0	141 (75.0)	441 (76.3)	1.00 (Reference)	1.00 (Reference)		
Deletion	*0/*0	47 (25.0)	137 (23.7)	1.07 (0.73-15.7)	1.08 (0.65-1.82)	0.718	
	missing	8 (4.3)	29 (5.0)				

*GSTT1*	*1/*1 + *1/*0	153 (81.0)	482 (82.5)	1.00 (Reference)	1.00 (Reference)		
Deletion	*0/*0	36 (19.0)	102 (17.5)	1.11 (0.73-1.69)	1.15 (0.66-2.02)	0.622	
	missing	7 (3.7)	23 (3.9)				

^†^Associations were determined using multivariate logistic regression models to estimate the risk of developing PCA using *GSTP1 *105 Ile/Ile, GSTM1 *1/*1 + *1/*0 and GSTT1 *1/*1 + *1/*0 as the reference genotypes.

^††^Risk estimates adjusted for age (continuous variable); prostate specific antigen (continuous variable); and West African Ancestry, *WAA *or admixture (continuous variable).

^†††^Differences in the frequency of high-risk and low-risk genotypes between cases and controls were determined using the chi-square test of association with a significance level of 0.05.

**Figure 1:**
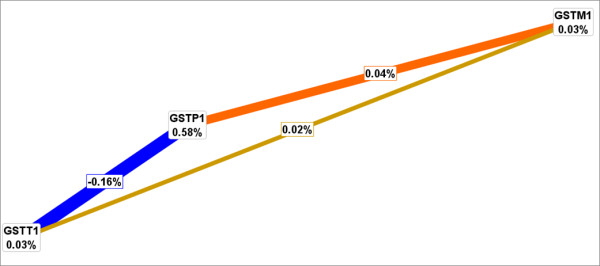
**Interaction entropy model for *GST *gene variations and prostate cancer risk**. This graphical model, describes the percent entropy that is explained by each *GST *SNP or a combination of two loci within our study population. Positive percent entropy indicates information gain or synergy. However, negative percent indicates redundancy or lack of information gain. Schematic coloration used in the visualization tools represents a continuum from synergy (i.e. non-additive) to redundancy. The colors range from red representing a high degree of synergy (positive information gain), orange a lesser degree, and gold representing independence and a midway point between synergy and redundancy. On the other hand, green represents redundancy. Note that the gene combination effect is primarily driven by the *GSTP1 *loci attributed to the lack of additional information gain comparing the *GSTM1*-*GSTP1 *axis (IG = 0.04%) to either *GSTP1 *or *GSTM1 *loci alone (IG = 0.58% and 0.03%, respectively)

We evaluated the independent effects of genetic variations in highly variant *GST *genes in relation to PCA susceptibility using unconditional LR models, as detailed in Table 2. Inheritance of at least one *GSTP1 *105 Val allele (linked with decreased capacity to conjugate electrophilic compounds) was associated with a 1.6-fold increase in prostate cancer risk (OR = 1.56; 95%CI = 0.95-2.58; p = 0.049). The *GSTP1 *Val loci was also identified as the best single factor predictor of PCA based on a cross-validation consistency of 100%, an average testing accuracy of 53% and a 1000-fold permutation testing p-value of 0.001 (Table 3). Upon evaluation of gene-gene combination effects, LR modeling revealed a 2.1-fold increase in PCA risk associated with men possessing the *GSTP1 *(Val/Val) and *GSTM1 *(*1/*1 + *1/*0) alleles (OR = 2.11; 95%CI = 1.07-4.16; p-value for interaction = 0.062). Initially, it appeared as though this interaction was a significant PCA predictor following MDR analysis with a statistically significant 1000-fold permutation testing p-value of 0.01. However, this gene combination effect was primarily driven by the *GSTP1 *loci attributed to the lack of additional information gain comparing the *GSTM1*-*GSTP1 *axis (IG = 0.04%) to either *GSTP1 *or *GSTM1 *loci alone (IG = 0.58% and 0.03%, respectively) (Figure 1). Lastly, no significant risk effects were detected among the *GST *deletion polymorphisms (p = 0.622) or the *GSTP1-GSTP1 *and *GSTM1-GSTT1 *combinations (p = 0.557 and 0.814, respectively). For our exploratory analysis of gene-environment interactions associated with risk, LR modeling indicated that smoking status did not modify the relationship between the *GST *SNPs and PCA risk (p for interaction ≥ 0.135), as shown in Table 4.

**Table 3:** Multifactor Dimensionality Reduction Models for *GST *gene variations and prostate cancer risk

Best Model	Cross Validation Consistency (CVC)	Average Testing Accuracy (ATA)	Permutation Testing p-value
One Factor			
*GSTP1*	10/10	0.530	0.001

Two Factor			
*GSTP1*	8/10	0.535	0.01
*GSTM1*			

**Table 4:** Combined effects of *GST *polymorphisms and smoking on prostate cancer risk

Gene	Polymorphism	Adjusted OR (95% CI) for Non-smokers^†‡^	Adjusted OR (95% CI) for Ever-smokers^††‡^	p-value for Interaction
*GSTP1*	AA (Ile/Ile) & AG (Ile/Val)	1.00 (Reference)	2.88 (0.49-16.82)	
A^313^G	GG (Val/Val)	1.91 (0.47-7.82)	1.42 (0.33-6.10)	0.928
Ile^105^Val				

*GSTM1*	*1/*1 + *1/*0	1.00 (Reference)	1.71 (0.59-4.97)	
Deletion	*0/*0	1.61 (0.36-7.28)	0.47 (0.11-2.09)	0.135

*GSTT1*	*1/*1 + *1/*0	1.00 (Reference)	1.03 (0.37-2.89)	
Deletion	*0/*0	1.02 (0.21-4.94)	2.12 (0.47-9.57)	0.410

^†^Associations were determined using multivariate logistic regression models to estimate the risk of developing PCA using *GSTP1 *105 Ile/Ile, GSTM1 *1/*1 + *1/*0 and GSTT1 *1/*1 + *1/*0 as the Reference genotypes.

^††^Risk estimates adjusted for age (continuous variable); prostate specific antigen (continuous variable); and West African Ancestry, *WAA *or admixture (continuous variable).

^‡^Non-smokers are subjects that have never smoked; Ever-smokers are subjects that are current or former smokers

## Discussion

Previous studies suggest that oxidative stress and reactive species are becoming increasingly important in prostate carcinogenesis[4,11,12,42-45]. If these electrophilic compounds are not removed or reduced to less active forms, they may result in damage to biomolecules (e.g., DNA, proteins, etc.) and ultimately lead to cellular dysfunction or transformation. Consequently, the role of antioxidant enzymes has been commonly investigated in relation to PCA development because of their function in the detoxification of potentially damaging carcinogens and/or reactive oxygen species (ROS)[4,46-48]. The *GSTs *in particular have been extensively studied since they are able to conjugate a wide range of oxidative substrates[6,11,43,49,50]. Variations within these genes can cause a loss or reduction in enzymatic activity and have been associated with increased risk of prostate as well as several other cancers (e.g., colon, breast, and lung)[6,12,51-53].

In the case of PCA, reports have observed that PCA cases possess lower antioxidant enzyme levels in prostate tissues compared to both healthy controls and men with benign prostatic hyperplasia (BPH)[48]. In addition, PCA tissues appear to contain higher amounts of ROS and oxidative DNA damage [46-48]. Some investigators speculate these effects may be attributed to *GST *genetic polymorphisms (i.e., GSTM1*0/*0, GSTT1*0/*0, and *GSTP1 *Val) linked with compromised oxidative repair capacity[5,12,26,41,43,54]. The goal of this study was to evaluate *GSTM1 *and *GSTT1 *gene deletions, variant *GSTP1 *105 Val, and cigarette smoking as predictors of PCA risk among 873 men of African descent (208 cases and 665 controls). We hypothesized that individuals who possess one or more genotypes associated with reduced detoxification would have a higher risk of developing PCA. In an exploratory analysis, we investigated the effects of cigarette smoking combined with these polymorphisms in relation to PCA risk. This increased risk may be partially attributed to exposure to tobacco carcinogens and altered capacity to metabolically detoxify hazardous compounds. Logistic regression analysis revealed a moderately significant association between PCA susceptibility and *GSTP1 *105 Val. These findings were confirmed using a robust data-mining tool specifically designed to evaluate main effects and higher order interactions. Although we did not detect any significant single gene effects among *GSTM1 *and *T1 *deletions, we did observe a moderately synergistic interaction between *GSTP1*-*GSTM1 *and PCA susceptibility. We found a 2.1-fold increase in PCA risk associated with men possessing 2-3 high risk alleles with the *GSTP1 *GG (Val/Val) &GSTM1 *1/*1 + *1/*0 genotype according to logistic regression analysis.

Numerous previous studies have investigated the role of these *GST *polymorphisms in relation to PCA susceptibility. For example, Agalliu et al. 2006 reported a moderate increase in PCA risk associated with the *GSTM1 *gene deletion for Caucasian men (OR = 1.54; 95%CI = 1.19-2.01); however, *GSTT1 *deletion and *GSTP1 *105 Val polymorphisms were not significantly associated with prostate cancer[5]. The null findings for *GSTT1 *and *GSTP1 *were corroborated in a recent meta-analysis of pooled data from ≥ 3,837 cases and ≥ 4,552 controls[12]. This same report, however, observed a 1.3 fold increase in PCA risk among all study participants was associated with the GSTM1*0 loci using pooled data from 4,564 cases and 5,464 controls (OR = 1.33; 95%CI = 1.15-1.55)[12]. Stratification based on self-identified race indicated that risk estimates were comparable between Caucasians and Asians; however, the *GSTM1 *and *GSTT1 *deletion polymorphisms did not modify risk among Africans or African-Americans[12]. Interestingly, only two published studies investigated the role of the *GSTP1 *105 Val SNP in relation to PCA among men of African descent[5,12,54]. The null findings in these two reports may be partially attributed to the failure to utilize rigorous statistical tools that permit the evaluation of main effects even in the presence of small sample sizes (i.e., at least 200 cases and 200 controls) such as MDR. The MDR selection of the *GSTP1 *105 Val loci as a significant PCA predictor in the current study is promising and will undergo subsequent validation within future studies among men of African descent.

We have considered the strengths and limitations of the current study. Although MDR doesn't allow for adjustments of covariates, it does control for multiple comparisons and spurious risk estimates by using a cross validation and permutation testing scheme as a built-in feature. Misclassification by case status is also a potential limitation for this study. There is a slight possibility that some of the controls with PSA less than 4 ng/ml may actually have prostate cancer that remains undetected. Controls who had an abnormal PSA (>4.0 ng/ml) and/or irregular DRE underwent multiple core needle biopsies. Patients with an abnormal biopsy were reclassified as cases. Participants with a PSA (i.e., ≤ 4.0 ng/ml) but an abnormal DRE were excluded abnormal DRE were excluded from participating in the current study. These individuals were excluded because we could not predict with any level of certainty whether these individuals would develop prostate cancer. We also excluded individuals who were: (1) diagnosed with BPH following a biopsy; or (2) had an abnormal PSA and/or an irregular DRE. Since one cannot predict with any level of certainty whether individuals diagnosed with BPH would develop prostate cancer, these individuals were excluded from the current study. As implied within Lobe et al., 2006, even after close inspection of prostate cancer tissue, it is possible to miss a microscopic nodule that can later develop into cancer[55]. If controls in our study population were still misclassified after undergoing a PSA test, DRE, and/or multiple core needle biopsies, then we may expect our calculated risk estimates to underestimate the relationship between the selected *GST *polymorphisms and prostate cancer susceptibility. But this issue plaques all cancer epidemiology studies. Unfortunately, it is impractical to subject all patients to a radical prostatectomy to permit an extensive evaluation and more accurate classification of case status.

Another challenge for genetic epidemiology studies involving study participants of African descent is their unique population history of gene flow from divergent populations[56,57]. The current study adjusted single loci models for genetic heterogeneity (i.e., population admixture). This also helps to circumvent misclassification of study participants related to self-identified race/ethnicity (SIRE). Our findings suggest that inclusion of WAA did not significantly change the risk estimates relative to unadjusted models; if anything it makes them more precise. Also, this study may be limited due to lack of consideration of the genetic heterogeneity of selected targets. This could be the reason we did not observe any statistical significant effects among *GST *polymorphisms and smoking interactions in relation to PCA risk (p = 0.105).

## Conclusion

In summary, our findings indicate a moderately significant association between risk and among men possessing at least one variant *GSTP1 *105 Val allele (p = 0.049) among men of African descent. MDR analysis validated the logistic regression findings and identified the *GSTP1*105 Val allele (p = 0.001) as the best single factor model for predicting PCA risk. The ability of MDR to evaluate main effects and remain effective with relatively small sample sizes of at least 200 cases and 200 controls strengthens the power of this study. Statistically significant single and multi-locus models maybe adjusted for potential confounders by placing the MDR output variables into a LR model. Furthermore, this study is robust since it utilizes multiple statistical analysis tools to examine the single gene as well as gene-gene or gene-cigarette smoking combination effects in relation to PCA risk.

## Competing interests

The authors declare that they have no competing interests.

## Authors' contributions

NAL, MLB, TTV and LRK designed and carried out genotyping assays and analyses; NAL, GNB, JHM and LRK performed statistical analyses; GNB, JHM, DWH and LRK served as mentors in designing and carrying out this project and editing the manuscript; All authors reviewed and edited the manuscript. All authors accepted the final manuscript.

## Pre-publication history

The pre-publication history for this paper can be accessed here:

http://www.biomedcentral.com/1471-2407/9/397/prepub
